# Analysis of the Clinicopathologic Characteristics and Prognosis of Head and Neck Lymphoma

**DOI:** 10.1155/2022/4936099

**Published:** 2022-02-22

**Authors:** Shufang Yan, Jiajia Ma, Meihong Yang, Bo Liu, Sijing Li, Liuqing Yang, Qian Zhang, Xinxia Li

**Affiliations:** ^1^Department of Pathology, The Tumor Hospital Affiliated to Xinjiang Medical University, No. 789 Suzhou Dongjie, Urumqi, The Xinjiang Uygur Autonomous Region of China, 830011, China; ^2^Xinjiang Medical University, No. 567 North Shangde Road, Urumqi, The Xinjiang Uygur Autonomous Region of China, 830011, China

## Abstract

Statistical reports on non-Hodgkin's lymphoma (NHL) of the head and neck combining clinical medicine with pathology are rare. To provide a basis for prognosis prediction and individualized treatment, we will investigate the clinicopathologic characteristics and prognosis of lymphoma in the head and neck region. Four hundred sixty-one patients with NHL in the head and neck region diagnosed through histological biopsy were retrospectively analyzed. Fluorescence in situ hybridization (FISH) and immunohistochemistry (IHC) were performed in all cases to evaluate the genetic status and protein expression levels. Patients were followed up by telephone. The prevalence rate of primary extranodal NHL (PENHL) in the head and neck region was 44.62% (166/372). The incidence of extranodal lymphoma accounted for 36.66% (169/461) of all head and neck lymphomas. Among the cases of PENHL of the head and neck, diffuse large B-cell lymphoma (DLBCL) (60/76, 78.95%) and extranodal NK/T-cell lymphoma, nasal type (ENKTCL) (21/24, 87.5%) were the most common subtypes originating from B-cell lymphoma (BCL) and T-cell lymphoma (TCL), respectively. The most common sites of nodal and extranodal onset were neck lymph nodes and the gastrointestinal tract, respectively. The most common and primary locations of BCL and TCL were the tonsils and nasal cavity, respectively. The 3-year survival rates of PENHL, ENKTCL, and DLBCL of the head and neck were 42%, 28.57%, and 41.67%, respectively, and the 5-year survival rates were 24%, 19.05%, and 20%, respectively. Survival analysis showed that male sex was a risk factor (HR = 5.421; 95% CI, 1.164-25.267; *p* < 0.05) and that comprehensive treatment was a protective factor (HR = 0.117; 95% CI, 0.025-0.545; *p* < 0.05) against extranodal DLBCL in the head and neck region. Bone marrow involvement was a risk factor for PENHL of the head and neck (HR = 5.072; 95% CI, 1.17-21.991; *p* < 0.05). The purpose of this review is to show that PENHL of the head and neck with high incidence deserves more attention, and a model of multidisciplinary diagnosis and treatment should be adopted.

## 1. Introduction

Lymphoma is a frequent hematological malignancy. In China, lymphoma has the highest incidence of the hemolymphatic system [1]. According to histopathology, lymphoma can be separated into two major categories: Hodgkin's lymphoma (HL) and non-Hodgkin's lymphoma (NHL). The subtypes of lymphoma are defined in accordance with the origin of the cells: B-cell lymphoma (BCL), T-cell (TCL) and natural killer-cell lymphoid tumors (T/NK-NHL), and HL [2]. NHL accounts for approximately 90% of all lymphomas [3]. Approximately 30% of NHLs originate from extranodal sites. Among the primary extranodal non-Hodgkin's lymphomas (PENHLs), the head and neck are often involved [2], and extranodal manifestations of head and neck lymphomas are different in the head and neck region and may occur in the major salivary glands, paranasal sinuses, mandible, maxilla, and Waldeyer's ring (largely depending on the specific NHL subtypes and frequently with their own characteristics). Judging by this, NHL of the head and neck region is a common disease in the process of clinicopathologic diagnosis. Due to a lack of adequate knowledge about this kind of disease and other reasons, it is often difficult to diagnose, and clinicians tend to miss it or misdiagnose it. In previous literature from home and abroad, most related studies on NHL of the head and neck are clinical analyses, but statistical reports on clinical medicine and pathology are rare. In our study, we reviewed epidemiological data, the clinical and pathological characteristics, treatment, and prognosis of the disease. The main purpose of this study was to summarize the incidence and features of extranodal NHL in the head and neck region from a single center in western China to provide a reference for the diagnosis and treatment of NHL.

## 2. Material and Methods

### 2.1. Clinical Data

A standardized medical history was obtained from all patients, including clinical examination results, age at diagnosis, sex, ethnicity, specific location of NHL in the head and neck, imaging evaluation (CT or B ultrasound), vital symptoms (B-symptoms: fever, night sweats, and weight loss), initial diagnosis time, known risk factors (infectious diseases such as HIV, hepatitis B, hepatitis C, tuberculosis, syphilis, and other systemic diseases, such as hypertension, diabetes, arrhythmia, and coronary atherosclerotic heart disease), histological biopsy results, and survival results. All patients finished the clinical examination (laboratory examination and histological biopsy) and underwent B-mode color Doppler ultrasonography of the neck when necessary. Some patients also underwent plain and enhanced CT scans of the head, neck, chest, abdomen, and pelvic cavity and a bone marrow biopsy. Blood tests included a complete blood count, and the following related parameters were recorded: white blood cell count, platelet count, and hemoglobin. Ann Arbor staging was performed in 100 patients with PENHL of the head and neck. Immunophenotyping based on Han's classification was used for patients diagnosed with diffuse large B-cell lymphoma (DLBCL). The aaIPI score and IPI score were determined according to age (≤60 years old and >60 years old), and pathological parameters such as the expression of proteins, rearrangement of genes, and positivity for Epstein–Barr virus (EBV) were recorded. During the diagnosis, all patients also underwent enhanced CT scans of the head, neck, chest, abdomen, and pelvic cavity or PET-CT and bone marrow biopsies. If central nervous system involvement was suspected, Ann Arbor staging also included contrast magnetic resonance imaging of the brain and/or spine. According to the 2013 Chinese guidelines for the diagnosis and treatment of DLBCL, the efficacy of the disease is divided into four stages: CR—complete remission, PR—partial remission, PD—progressive disease, and SD—stable disease.

### 2.2. Pathological Data

#### 2.2.1. Immunohistochemistry (IHC)

Using a fully automatic immunohistochemical instrument, antibodies suitable for paraffin sections were chosen. The main antibodies used and their working concentrations are shown in Table 1. Among them, the cutoff scores for MYC and Bcl-2 overexpression were ≥40% and ≥70%, respectively, and the positive standards for CD10, Bcl-6, and MUM1 were ≥30% [4]. Double-expressor lymphoma (DEL) refers to simultaneous expression of MYC/Bcl-2 protein (DEL-MYC/Bcl-2), and triple-expressor lymphoma (TEL) refers to simultaneous expression of MYC/Bcl-6/Bcl-2 protein (TEL-MYC/Bcl-2/Bcl-6) [5] in lymphoma patients. DLBCL was classified according to Han's algorithm, where the germinal center B cell (GCB) subtype had a CD10+ or CD10-, Bcl-6+, and MUM1- phenotype, whereas the Bcl-6- or Bcl-6+, CD10-, and MUM1+ phenotype represented the non-GCB subtype (including ABC subtype and unclassified subtype in this study).

### 2.3. Fluorescence In Situ Hybridization (FISH)

FISH was performed on 60 DLBCL formalin-fixed paraffin-embedded (FFPE) samples following the manufacturer's instructions. Detection of MYC, Bcl-2, and Bcl-6 gene abnormalities and two-color separation and rearrangement probe (including Bcl-6, MYC, and Bcl-2) kits were purchased from the Abbott Molecular Biology Company. Samples with MYC, Bcl-2, and Bcl-6 segregation were used as positive controls, and normal lymph nodes were used as negative controls. The FISH signal pattern of the separation probe in normal cells consists of two yellow signals or tightly located red (the organ color shows red on the purple background of DAPI) and green signals, and the distance between them is less than two signal sizes. Cells with a yellow, green, and red signal with a distance of ≥2 signal sizes were defined as positive for gene rearrangement, with three separate probes. The probe signals for a monolayer of at least 200 tumor cell nuclei were counted per sample at 100x magnification, and genetic alterations were determined when they exceeded a 10% threshold in the number of nuclei. FISH V.2.0 software was used to analyze the results.

### 2.4. In Situ Hybridization

An EBER (EBV-encoded RNA) *in situ* hybridization kit was purchased from ZSBIO. The specific steps were performed according to the manufacturer's instructions, and nasopharyngeal carcinoma tissues with positive EBER were treated as a positive control.

### 2.5. Follow-Up Visits

The time of diagnosis was when the follow-up visits began, and the deadline was August 19, 2020. The patient follow-up visits were held at the hospital or via telephone. The reasons for termination of the follow-up (such as death due to illness, loss to follow-up, or other irrelevant reasons) were recorded. Total survival time (TST) was defined as the time from the date of pathological diagnosis to the time of death or last follow-up. The median survival time (MST) is also known as the half-life, and a cumulative survival rate of 0.5 is the corresponding survival time. Overall survival (OS) is the ratio of the total number of survivors at the end of the entire follow-up period to the total number of people initially observed. The survival time of the patients who died within 1 month after the initial diagnosis was recorded as “0.”

### 2.6. Statistical Analysis

The experimental data were processed using SPSS 23.0 statistical software. Unpaired *t* tests were used for measurement data, and differences between different groups were compared using the *χ*^2^ test or Fisher's exact test. Survival curves were drawn according to the Kaplan–Meier method in univariate survival analysis, and the log-rank test was used to compare survival rates among various groups. A Cox regression model was applied to multivariate survival analysis, and the bilateral 95% CI was treated as the statistical standard. A *p* value of <0.05 was considered statistically significant.

## 3. Results

### 3.1. Epidemiology and Characteristics of the Head and Neck Cohort

**Figure 1:**
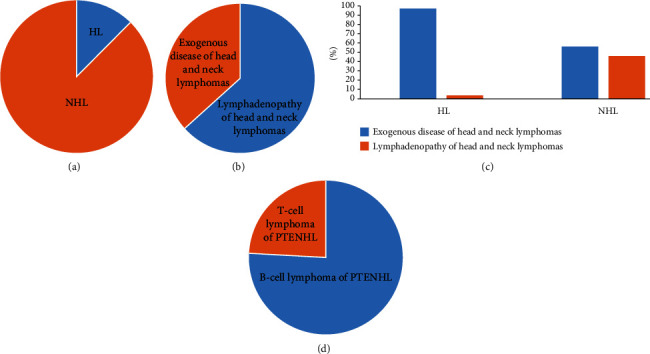
Clinical characteristics of extranodal non-Hodgkin's lymphoma in the head and neck region: (a) proportion of all lymphomas; (b) lymphadenopathy or exogenous disease proportion of head and neck lymphoma; (c, d) two major categories and two subtypes of PENHL in the head and neck region. PENHL: primary extranodal non-Hodgkin's lymphoma; HL: Hodgkin's lymphoma; NHL: non-Hodgkin's lymphoma.

One thousand twenty-nine patients with lymphoma diagnosed through histological biopsy at the Tumor Hospital Affiliated with Xinjiang Medical University from January 2010 to July 2020 were retrospectively analyzed, including 128 HL (extranodal: 118; nodal: 10) and 901 NHL (extranodal: 559; nodal: 342) (Figure 1(a)). The most common sites of nodal onset and extranodal onset were neck lymph nodes and the gastrointestinal tract, respectively. In this study, for the first time, 461 patients with head and neck lymphomas diagnosed by histological biopsy were retrospectively analyzed. The number of patients with lymphadenopathy was 292, and the incidence of extranodal disease was 169, accounting for 63.34% (292/461) and 36.66% (169/461) of head and neck lymphomas, respectively (Figure 1(b)). Finally, 89 cases of HL were screened: the number of extranodal patients was 3, and the number of nodal patients was 86. Three hundred seventy-two cases of NHL were selected: extranodal: 166, 44.62%; nodal: 206, 55.38% (Figure 1(c)). Among the 372 NHL patients, DLBCL accounted for 172 cases: seventy-five extranodal patients were included, and 97 nodal patients were included in this study, accounting for 43.61% and 56.39% of all DLBCL cases, respectively. Among the 166 extranodal NHL patients, 66 cases of NHL were removed due to incomplete extranodal NHL data; finally, 100 cases were included in this study. Among the 100 patients with primary extranodal NHL in the head and neck region, 59 were males and 41 were females, aged between 2 and 87 years; the median age was 58, and 58 cases were ≥50 years old. There were 5 cases of bone marrow involvement and 21 cases of multiple site involvement. This study included 67 Han nationalities and 33 ethnic minorities.

Among the 100 patients with primary extranodal NHL in the head and neck region (Figure 1(d)), 76 cases of B-cell lymphoma were identified: sixty cases of DLBCL, six cases of mucosa-associated lymphoid tissue extranodal marginal zone lymphoma (MALT), three cases of follicular lymphoma (FL), three cases of Burkitt lymphoma (BL), one case of B small lymphoid cell lymphoma/chronic lymphocytic leukemia, two cases of B-lymphoblastic lymphoma (B-LBL), and one case of mantle cell lymphoma. DLBCL was the most common subtype with a median age of 58 and an average age of 55.88. Twenty-four cases of T-cell lymphoma were identified: twenty-one cases of extranodal NK/T cell lymphoma, nasal type (ENKTCL); two cases of peripheral T-cell lymphoma (PTCL); and one case of anaplastic large cell lymphoma (ALCL) with positive ALK. ENKTCL accounted for the highest ratio, which included 13 males and 8 females; the ratio of males to females was 1.6, and the median age was 49. The most common locations of primary BCL and TCL were the tonsils and nasal cavities, respectively (Table 2).

Among the 31 patients with NHL originating from the nose, 10 cases arose from the nasopharynx, whereas the remaining cases involved the nasal cavity. There were 17 cases of primary ENKTCL, three cases of DLBCL, and one case of PTCL. Twenty-seven patients had B-symptoms, 16 patients had abnormal hemograms, 28 patients had no recorded International Prognostic Index (IPI) score, five patients received IPI scores but did not have improved IPIs, and nine patients were still incomplete after rescoring (Table 2).

### 3.2. Immunophenotypic Features

**Figure 2:**
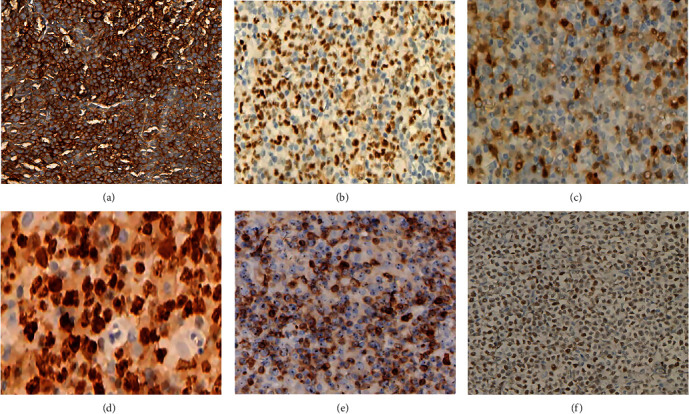
Immunohistochemical results (EnVision method, original magnification 200x) of DLBCL. (a–f) CD10, Bcl-6, Mum1, Ki67, Bcl-2, and MYC expression on oncocytes.

BCL: sixty cases of DLBCL were identified. The expression rates of CD10, Bcl-6, CD20, Mum1, Ki67 index ≥ 70%, Bcl-2, and MYC were 18.33% (Figure 2(a)), 41.67% (Figure 2(b)), 100%, 60% (Figure 2(c)), 45% (Figure 2(d)), 20% (Figure 2(e)), and 18.33% (Figure 2(f)), respectively. There were 23 cases of GCB type, 34 cases of ABC type, and three cases of unclassified DLBCL. Six cases of DE lymphoma and five cases of TE lymphoma were included. Six cases of MALT were identified: the expression rates of CD20, CD43, PAX5, and Bcl-2 were 100%, 25%, 60%, and 100%, respectively. Three cases of FL were identified: the expression rates of Bcl-2 and CD20 were 66.67% and 100%, respectively. Three cases of BL were identified: the expression rates of Bcl-6, CD10, CD20, CD79a, and Ki67 index ≥ 70% were 100%. Two cases of B-LBL were identified: the expression rates of CD10, CD20, CD79a, PAX5, and TdT were 100%. One was B small lymphoid cell lymphoma/chronic lymphocytic leukemia: the expression rates of CD5, CD23, CD20, and CD79a were 100%, and the Ki67 index was ≥40%. One case of MCL was identified: the expression rates of Cyclin D1, CD5, CD20, and CD79a were 100%, and the Ki67 index was ≥60%.

**Figure 3:**
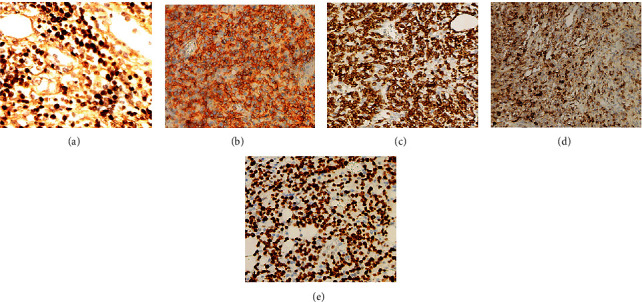
Immunohistochemical results (EnVision method, original magnification 200x) of extranodal NK/T cell lymphoma, nasal type (ENKTCL): (a) EBER-positive oncocytes; (b–e) CD56, CD3, GrB, and Ki67 expression on oncocytes. EBER: Epstein–Barr virus-encoded RNA.

**Figure 4:**
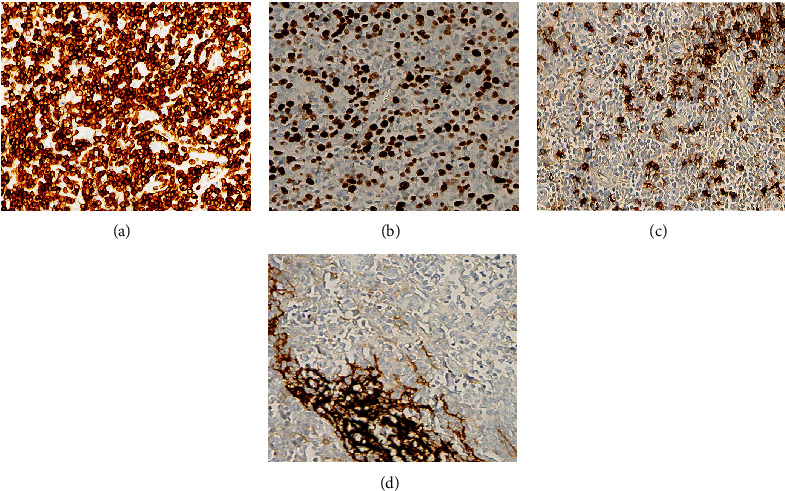
Immunohistochemical results (EnVision method, original magnification 200x) of peripheral T-cell lymphoma: (a, b) CD3 and Ki67 expression on oncocytes; (c, d) CD20- and CD23-negative oncocytes.

TCL: twenty-one cases of ENKTCL were identified. The expression rates of EBER, CD20, CD79a, CD56, CD3, CD4, CD8, GrB1, TIA-1, CD43, and Ki67 ≥ 60% were 100% (Figure 3(a)), 5.88%, 42.86%, 61.11% (Figure 3(b)), 100% (Figure 3(c)), 71.43%, 60%, 75% (Figure 3(d)), 93.75%, 66.67%, and 28.57% (Figure 3(e)), respectively. One case was ALCL with ALK positivity: the expression rates of ALK, CD30, CD2, CD3, and EMA were 100%. Two cases of PTCL were identified: the expression rates of CD3 (Figure 4(a)) and Ki67 (Figure 4(b)) index ≥ 70% were 100%. The expression rates of CD20 (Figure 4(c)) and CD23 (Figure 4(d)) were negative.

In the above 100 cases, due to incomplete tests of MYC, Bcl-2, or Bcl-6, DE and TE could not be acquired completely.

### 3.3. Cytogenetic Analysis

**Figure 5:**
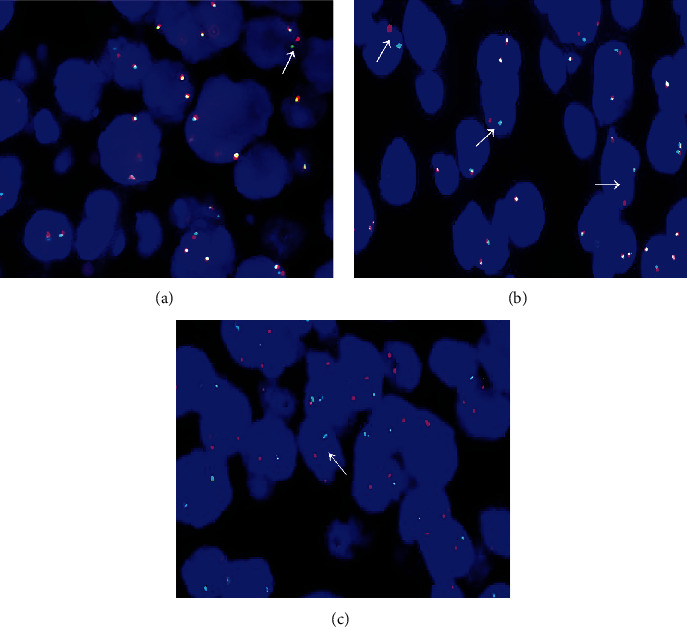
Representative FISH findings in patients with B-cell lymphoma. (a–c) Shows Bcl-2, Bcl-6, and MYC rearrangements. FISH: fluorescence in situ hybridization (×1000).

Out of the 60 patients with DLBCL, 33 finished TH or DH, no patient was confirmed with TH, and two patients accounting for 3.33% (2/60) of the patients with DLBCL had double-hit lymphoma (DHL). All of them had DHL*-*MYC/Bcl-6, including 1 case of DHL combined with TEL (Figures 5(a)–5(c)).

### 3.4. Clinical Outcomes

#### 3.4.1. Follow-Up and Ann Arbor Staging

During a follow-up of 240 months, 100 patients were followed up systematically. The MST was 24 months (1 to 240 months), and the average follow-up time was 37 months. Seven patients were lost to follow-up, and 21 died during the follow-up period. Among the patients with B-cell lymphoma (DLBCL), three died in the early stage (stages I-II), and 10 died in the late stage (stages III-IV). One patient with MALT died in the late stage and lived for 11 months. Among the patients with T-cell lymphoma (ENKTCL), one died in the early stage, and five died in the late stage. During the follow-up, three patients who had been confirmed to be in PR still complained of yellow, pungent pus coming out of their nostrils. One ENKTCL patient with positive CD20 was a 73-year-old elderly male with occasional fatigue complicated with hypertension and Ki67 > 5%. In this patient, PR was achieved after comprehensive treatment (radiotherapy or chemotherapy or chemoradiotherapy combined with surgery and/or transplantation), TST was 105 months, the original focus was reduced by more than 50%, and no new or metastatic lesions were found in CT reexamination. One patient with EBER-positive PTCL died in the late stage after living for 40 months. The other patient was a middle-aged female with Ki67 > 85%, a submental primary site, PD after chemotherapy, and TST of 11 months.

### 3.5. Treatment Method and Therapeutic Effect Analysis

#### 3.5.1. Patients with B-Cell Lymphoma

Sixty patients with DLBCL were found: thirty-three patients finished EBV testing: two were positive for EBV, and the MST was 51 months. Thirty-one were negative for EBV, and the MST was 24 months. Thirty-four cases of ABC type were detected: nine patients died, with an MST of 14 months. Four cases were lost to follow-up. Twenty-one cases survived, with an MST of 45.5 months. Twenty-three cases of GCB type were detected: three patients died with an MST of 15 months. Twenty cases survived with an MST of 44 months. Three cases of unclassified DLBCL type were detected: one patient died with a TST of 40 months, and two patients survived. The 3-year and 5-year survival rates of DLBCL were 41.67% and 20%, respectively, and there was a significant difference in the 3-year and 5-year mortality rates (*χ*^2^ = 6.604, *p* < 0.05).

Six cases of MALT were detected: five were in early stage and one was in the late stage. One died and five survived. Two patients were complicated with an autoimmune disease called Sjogren's syndrome, and their MST was 32.5 months. To slow down renal dysfunction, one patient with a history of kidney failure used chemotherapy only once, and then, the patient did nothing about kidney failure and lymphoma at the same time. Ultimately, the two diseases reinforced each other, and the patient died after a TST of 40 months; the primary site was located in the nasopharynx. The other one was an elderly female. The primary site was in the parotid gland, she achieved PR after comprehensive treatment, survived until the end of follow-up, and her TST was 25 months. The remaining four survived after treatment. After chemotherapy, two cases with an MST of 35.5 months achieved CR, 3 cases were in the oral cavity. Three cases of FL were found, with a male/female ratio of approximately 1 : 1.5. There was no bone marrow involvement at the initial diagnosis, one was in the early stage, two were in the late stage, the median age was 56, the average survival time was more than 5 years, 2 achieved CR after chemotherapy, 1 achieved SD after chemotherapy, the MST was 64 months, and all survived at the end of follow-up. One case of B small lymphocytic lymphoma/chronic lymphocytic leukemia was found in an elderly male who had a Ki67 > 40%, the primary site was the mandible, the TST was 76 months, and PR was achieved after chemotherapy. Three cases of BL were found: a female child, a young male, and an elderly male, with a median age of 29 years; Ki67 was close to 100%; all were in the late stage; and the primary sites were the maxilla, gingiva, and tongue root. Two cases were SD after chemotherapy, one case reached PR, and the MST was 41 months. Two cases of B-LBL were found: all were male children with a median age of 6.5 years, the primary sites were the preauricular position and parotid gland, one achieved PR after chemotherapy, and the other achieved SD after comprehensive treatment. The MST was 41 months. One case of MCL was found in an elderly male with Ki67 > 50%, the primary site was the mandible, the patient achieved CR after comprehensive treatment, and the TST was 95 months.

#### 3.5.2. Patients with TCL

Among 21 patients with ENKTCL, three patients were lost to follow-up, twelve survived, and six died. Among the deceased patients, the MST was 9.5 months, one was complicated with hepatitis B, and three cases were located in the nasal cavity and two in the laryngopharynx, of which two patients developed hemophagocytic syndrome and eventually died with pancytopenia and abnormal liver function. One received comprehensive treatment, multiple chemotherapy schemes were changed frequently from the beginning of treatment, the disease progressed continuously, and the TST was 41 months. Another accepted not only continuous chemotherapy but also frequent changes in chemotherapy regimens; owing to the rapid PD, the TST was 12 months. Sixteen patients had completed EBV tests, and all of them had positive results. Five patients did not finish the EBV tests. The number of patients with primary sites in the nasal part and laryngopharynx was 19 and 2, respectively. The 3-year and 5-year survival rates of ENKTCL were 28.57% and 19.05%, respectively, but there was no difference in the 3-year and 5-year mortality composition (*χ*^2^ = 0.525, *p* > 0.05).

One case of ALCL with positive ALK was a young male with Ki67 > 90%, the primary site was the mandible, CR was achieved after chemotherapy, and the TST was 8 months. One case of PTCL was seen: the patient was a young female with a primary site of the nasal cavity who achieved CR after comprehensive treatment. The TST was 240 months. She was 20 years old and survived until the end of follow-up. One case of PTCL was seen: a middle-aged female with the primary site of the chin, who died after a follow-up of 11 months.

A significant difference was seen in the positive rate of EBV between the ENKTCL and DLBCL groups (*χ*^2^ = 65.304, *p* ≤ 0.001). For the ENKTCL patients, the 3-year and 5-year survival rates were shorter than those of the DLBCL patients, but there was no difference in the 3-year (*χ*^2^ = 1.129, *p* > 0.05) and 5-year mortality rates (*χ*^2^ = 0.009, *p* > 0.05). The 3-year and 5-year survival rates of primary extranodal NHL in the head and neck region were 42% and 24%, respectively, and there was a significant difference in the 3-year and 5-year mortality rates (*χ*^2^ = 7.327, *p* < 0.05).

### 3.6. Survival Analysis

#### 3.6.1. Univariate Survival Analysis

**Figure 6:**
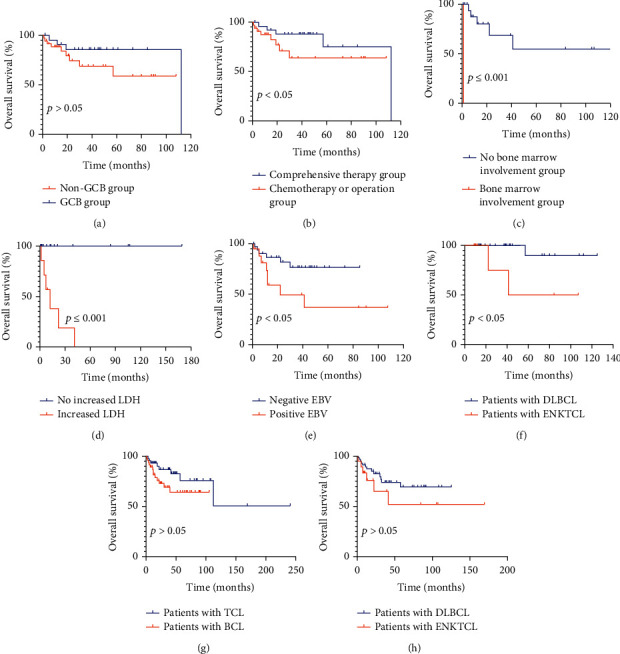
Univariate analysis of patients with primary extranodal non-Hodgkin's lymphoma in the head and neck region among various groups. (a) The prognosis of patients with GCB type was better than that of patients with non-GCB subtype. (b) Comprehensive treatment and operation or chemotherapy was related to the OS of DLBCL patients. (c, d) For patients with ENKTCL, bone marrow involvement and increased LDH were related to OS. (e) The OS of patients with positive EBV was lower than that of the negative patients. (f) When comprehensive treatment was applied, the OS of patients with DLBCL was higher than that of patients with ENKTCL. (g) There was no difference in OS between patients with T-cell lymphoma and B-cell lymphoma. (h) There was no difference in OS between patients with DLBCL and ENKTCL. LDH: lactate dehydrogenase; ENKTCL: extranodal NK/T-cell lymphoma, nasal type; DLBCL: diffuse large B-cell lymphoma.

Among the DLBCL patients, the prognosis of patients with the GCB type was better than that of patients with the non-GCB subtype (*χ*^2^ = 2.127, *p* > 0.05) (Figure 6(a)). Comprehensive treatment and operation or chemotherapy were related to the OS of DLBCL patients (*χ*^2^ = 7.885, *p* < 0.05) (Figure 6(b)). Sex, age greater than or equal to 50 years, ethnicity, multiple site involvement, bone marrow involvement, primary site, combined systemic disease, and increased LDH and B-symptoms were not related to the OS of DLBCL patients. For patients with ENKTCL, bone marrow involvement (*χ*^2^ = 20.000, *p* ≤ 0.001) (Figure 6(c)) and increased LDH (*χ*^2^ = 14.492, *p* ≤ 0.001) (Figure 6(d)) were related to OS. The OS of patients with positive EBV was lower than that of negative patients (*χ*^2^ = 4.691, *p* < 0.05) (Figure 6(e)). When comprehensive treatment was applied, the OS of patients with DLBCL was higher than that of ENKTCL patients (*χ*^2^ = 5.400, *p* < 0.05) (Figure 6(f)). There was no difference in OS between patients with B-cell lymphoma and T-cell lymphoma (*χ*^2^ = 2.053, *p* > 0.05) (Figure 6(g)). There was no difference in OS between patients with DLBCL and ENKTCL (*χ*^2^ = 1.358, *p* > 0.05) (Figure 6(h)). In the head and neck region, there was no difference in OS between patients with DLBCL and PENHL (*χ*^2^ = 0.028, *p* > 0.05), and no difference was seen between patients with ENKTCL and PENHL (*χ*^2^ = 1.365, *p* > 0.05).

#### 3.6.2. COX Multivariate Survival Analysis

Male sex was a risk factor (HR = 5.421; 95% CI, 1.164-25.267; *p* < 0.05), and comprehensive treatment was a protective factor (HR = 0.117; 95% CI, 0.025-0.545; *p* < 0.05) for extranodal DLBCL in the head and neck region. Bone marrow involvement was a risk factor for extranodal NHL in the head and neck region (HR = 5.072; 95% CI, 1.17-21.991; *p* < 0.05).

## 4. Discussion

Lymphoma is the third most frequent malignancy in the world, accounting for 3% of all malignant tumors. Lymphoma accounts for 12% of all malignant tumors of the head and neck and is the third largest malignant tumor after squamous cell carcinoma and thyroid cancer [2, 6, 7]. In recent years, the incidence of NHL in China has tended to advance [8, 9]. This study shows that in the past 20 years, the extranodal incidence of all head and neck lymphomas was 36.66% (169/461), which was lower than that reported (57% and 50%, respectively) [2]. The most common site of onset was the neck lymph nodes, followed by the gastrointestinal tract, which was consistent with a previous report [10]. This may be related to some patients who go to other hospitals in this area. The distribution of extranodal and nodal lymphomas and the primary sites of lymphomas was consistent with previous research [10]. Consistent with a previous study [11, 12], the prognosis of patients with early-stage disease was better than that of patients with late-stage disease, and improving the detection of EBV and making a clear diagnosis in time were beneficial to patients. This may be combined with systemic disease and explains why DLBCL patients have higher mortality. When the patient was a male with DLBCL or a patient was diagnosed with NHL combined with bone marrow involvement, it indicated a poor prognosis.

### 4.1. Patients with BCL

Consistent with the literature [13, 14], DLBCL accounted for the largest proportion of extranodal NHL of the head and neck. DLBCL can occur in patients at any age, but the majority of them are middle-aged and elderly patients, which is consistent with the results of this study, and the incidence of extranodal disease represents approximately 40% [15], a result that was similar to that of this study. MYC, Bcl-2, and/or Bcl-6 rearrangement and protein expression were identified as prognostic factors for DLBCL [16, 17]. This study indicates that immunohistochemistry plays a vital role in forecasting prognosis. Pathologists should improve DEL, TEL, and FISH examinations as soon as possible to discover high-grade B-cell lymphoma with poor prognosis. The number of cases of THL was lower and the number of cases of DHL was higher than reported [4]. It may be associated with the short prefix time of the specimen, which was applied so that the protease was easy to digest and the hybridization site was fully exposed. Clinical studies have shown that EBV-positive DLBCL is considered to be a new NHL, representing 30-40% of cases of NHL [18]. Considering the number of cases, the MST was longer than that of EBV-negative cases, which was inconsistent with the literature [12]. Consistent with a previous report [19], patients with non-GCB type have a worse prognosis than those with GCB type. Regarding age and DLBCL prognosis, continuous controversy has existed [20, 21]. Perhaps for Chinese patients, the age of initial diagnosis over 60 years old will indicate a poor prognosis, which may be related to the Chinese diet, physique, heredity, and other factors. These findings need to be confirmed by further research. During our follow-up, we found that although some patients underwent autologous hematopoietic stem cell transplantation (ASCT) in later stages, the effect of final treatment was poor because the best time for transplantation was missed. Whether sex and B-symptoms are related to the TST of DLBCL still needs to be studied using multicenter and large samples. The 3-year survival rates were lower than those reported in the literature, which may be related to completely resected tumors [22]. MALT can be further divided into gastrointestinal MALT and nongastrointestinal MALT. In the early stage, this lesion is called “pseudolymphoma” [23]. Two cases were complicated with Sjogren's syndrome, which was consistent with a previous report [24]. Five survived until the end of follow-up, and the disease appeared in different places, which once again confirmed that MALT develops slowly and is an inert lymphoma that can occur in many sites. The MALT type is a rare extranodal NHL subtype in the head and neck region, and it mainly occurs in the oral cavity, which is consistent with a previous report [25]. FL is a malignant lymphoma originating from B-cells in the follicular germinal center with an upward trend. A median age of 56 and a male-to-female ratio of 1 : 2 were consistent with the results of a related study [26]. An average survival time of more than 5 years was consistent with a previous research [27]. No case included partial transformation to DLBCL and bone marrow involvement, which was inconsistent with a previous report [28], considering fewer cases. BL is a kind of NHL originating from GCB. The Ki67 being close to 100% is consistent with this study [29]. Additionally, this study showed that BL was diagnosed initially at a higher stage, which was easily overlooked, and we should pay more attention to it to choose an appropriate treatment strategy to improve the therapeutic effect. BL is classified as endemic, sporadic, and immunodeficient [15]. The primary sites, which were the maxilla, gingiva, and tongue root, were not consistent with those reported [30]. After chemotherapy, all patients survived until the end of follow-up, and the expression of proteins was consistent with that mentioned in a previous report [31].

According to this study, the majority of BCLs in the head and neck regions are DLBCLs. We should place importance on DLBCL. Therefore, doctors in hematology, oncology, and pathology departments should collaborate with each other in their day-to-day work and try their best to advocate for ASCT in time to improve the prognosis. On the other hand, the diagnosis and treatment of lymphoma require multidisciplinary cooperation.

### 4.2. Patients with TCL

In China, ENKTCL is one of the most common subtypes of NHL [32] and is closely related to EBV infection [33]. EBV infection present in the occurrence of ENKTCL was confirmed once again. The 3-year and 5-year survival rates were lower than those reported in previous reports [34, 35]. Some ignored the seriousness of refusing to accept treatment at the initial stage, which then resulted in increasing mortality. Consistent with previous reports [15, 36], the most common site of ENKTCL and the primary site with the highest incidence of NHL were the nasal cavity. The histologic type with the highest incidence was DLBCL, followed by ENKTCL, which was not consistent with a previous report [36]. Xinjiang is a multiethnic region, and the above result may be related to the fact that there were 33 minority cases. Ethnic and individual differences may lead to a high incidence of DLBCL. In this group of ENKTCL patients, the mortality rate and MST were lower than those reported in the literature [35], so attention must be paid to these patients. Two patients developed hemophagocytic syndrome: one received comprehensive treatment, 1 received chemotherapy, and 2 died of rapid PD. Therefore, when the patient has hemophagocytic syndrome, the condition is critical, the prognosis is extremely bad, and the mortality is high. The positive rate of CD56 was 61.11%, which is a lower ratio than that reported [37], considering the lower number of cases of ENKTCL in this group. Consistent with a report [38], one patient with CD20 positivity in this group was a 73-year-old male, but inconsistent with that report, the Ki67 was >5%, and there was low invasiveness and inert clinical progress, as well as accompanying hypertension and occasional fatigue. Increased LDH and bone marrow involvement relevant to OS of ENKTCL were consistent with those reported in a previous study [39].

This study suggests that clinicians usually pay attention to screening EBV-positive patients. In combination with assessing the clinical symptoms, further examination should be recommended if necessary to help doctors exclude ENKTCL as soon as possible. Focusing on the hemogram may mislead the clinician. Previously, patients' chief complaints and medical histories were vital. Observing the dynamics of disease development is indispensable. Biopsy surgeons should strive to obtain a sufficient amount of pathological tissue. Additionally, if there are no clear tumor cells in the first biopsy but a large area of coagulative and necrotic tissue can be seen, it is necessary to perform another tissue biopsy or close follow-up. Fast and accurate pathological diagnosis is a required condition to improve the effect and prolong the lifespan of patients with ENKTCL.

B-symptom is the main prognostic factor [35]. In this study, 27 patients had B-symptoms, which was higher than previously reported [40]. Weight loss, which is relatively subjective, may result in false-positives for B-symptoms. Considering the problem of credibility, clinicians should not judge the disease only by B-symptoms, which may mislead the doctor. Only one patient suffering from HIV was diagnosed with DLBCL, which was inconsistent with a previous report [41]. Among the BL patients who were found, two were males, as reported [42], and none of the cases were associated with HIV or EBV, which was inconsistent with the report [41]. Furthermore, consistent with previous reports, PTCL and B small lymphocytic lymphoma/chronic lymphocyte leukemia were discovered [13, 31]. MCL [43] and lymphoblastic lymphoma [31] are rare subtypes. It has been reported that plasmoblastoma can be seen, but it was not seen in this study [31].

## 5. Conclusions

This study showed that the incidence of primary extranodal NHL in the head and neck region was high. Simultaneously, we were not able to apply methods that are useful for guiding clinical diagnosis and treatment until we knew more about the pathology, such as the results of the FISH and IHC tests. To control the progression of NHL in the head and neck, new research methods for tumor prevention, diagnosis, and treatment are necessary. This study reminds otolaryngologists, hematologists, and oncologists to combine clinical disciplines with pathology and pathophysiology, which is beneficial for doctors to understand the pathological and pathophysiological mechanisms of the disease and may be the next step for clinicians and pathologists to explore.

## Figures and Tables

**Table 1:** Primary antibodies and conditions used for immunohistochemical staining.

Antigen	Clone	Source	Dilution	Positive location
ALK	SP8	ZSBIO	1 : 80	Nuclei/cytoplasm
Bcl-2	100/D5	Shanghai Long Island	1 : 100	Cytoplasm
Bcl-6	GI191E/A8	ZSBIO	1 : 80	Nuclei
CD3	UCHT1	Shanghai Long Island	1 : 50	Cytoplasm/cytomembrane
CD5	SP19	ZSBIO	1 : 100	Cytomembrane
CD8	SP16	ZSBIO	1 : 100	Cytomembrane
CD10	56C6	Gene Company	1 : 50	Cytomembrane
CD20	L26	Dako	1 : 150	Cytomembrane
CD30	Ber-H2	MXBIO	1 : 100	Cytomembrane/cytoplasm
CD43	DF-T1	ZSBIO	1 : 100	Cytomembrane/cytoplasm
CD45RO	UCHL1	Dako	1 : 100	Cytomembrane
CD56	IB6	Gene Company	1 : 100	Cytomembrane
CD79a	HM47/A9	Shanghai Long Island	1 : 100	Cytomembrane
Cyclin D1	SP4	Gene Company	1 : 80	Nuclei
EMA	GP1.4	Shanghai Long Island	1 : 200	Cytomembrane
Granzyme-B	GZB01	MXBIO	1 : 50	Cytoplasm
MUM1	Mum1p	MXBIO	Ready to use	Nuclei
MYC	Y69	ZSBIO	1 : 150	Nuclei
Ki67	MIB-1	Gene Company	1 : 150	Nuclei
Perforin	MRQ-23	ZSBIO	1 : 20	Cytoplasm
TIA-1	2G910F5	ZSBIO	1 : 100	Cytoplasm

**Table 2:** Frequency of histologic types of head and neck lymphoma including epidemiology, symptomatology, disease manifestations, localization, and laboratory findings.

Item	B-cell lymphoma	T-cell lymphoma	*p* value
*n*	76	24	—
Age	—	—	0.186
Mean ± SD	54.37 ± 19.59	48.54 ± 15.45	—
Median	58	48	—
Sex, *n* [%]	—	—	0.939
Male	45 [59.21; 45]	14 [58.3; 14]	—
Female	31 [40.78; 31]	10 [41.7; 10]	—
Ethnicity, *n* [%]			0.591
Han	52 [68.42; 52]	15 [62.5; 15]	—
Minorities	24 [31.58; 24]	9 [37.5; 9]	—
Primary localization, *n* [%]	—	—	0.000∗
Nasopharynx	8 [10.53; 8]	2 [8.33; 2]	—
Nasal cavity	4 [5.26; 4]	17 [70.83; 17]	—
Tonsil	22 [28.95; 22]	0 [0]	—
Mandible	14 [18.42; 14]	1 [4.17; 1]	—
Parotid gland	6 [7.89; 6]	0 [0]	—
Laryngopharynx	4 [5.26; 4]	3 [12.5; 3]	—
Tongue	4 [5.26; 4]	0 [0]	—
Others	14 [18.42; 14]	1 [4.17; 1]	—
Laboratory parameter, mean ± SD	—	—	—
Leucocytes	6.84 ± 3.28	7.18 ± 5.07	0.697
Hemoglobin	125.22 ± 22.30	117.92 ± 16.86	0.143
Thrombocyte	236.60 ± 89.41	236.33 ± 95.54	0.990
LDH increased	21 [27.63; 21]	8 [33.33; 8]	1.000
Systemic disease, *n* [%]	33 [43.42; 33]	4 [16.67; 4]	0.024∗
Communicable diseases, *n* [%]	9 [10.53; 8]	1 [4.17; 1]	0.343
Hepatitis B	7 [9.21; 7]	1 [4.17; 1]	0.427
HIV	2 [2.63; 2]	0 [0]	0.422
Others	24 [31.58; 24]	3 [12.50; 3]	0.066
Histologic subtype, *n* [%]	76	24	—
DLBCL	60 [78.95; 60]	—	—
MALT	6 [7.89; 6]	—	—
FL	3 [3.95; 3]	—	—
BL	3 [3.95; 3]	—	—
B small lymphoid cell lymphoma/chronic lymphocytic leukemia	1 [1.32; 1]	—	—
B-LBL	2 [2.62; 2]	—	—
MCL	1 [1.32; 1]	—	—
ENKTCL	—	21 [87.50; 2]	—
PTCL	—	3 [12.50; 3]	—
B-symptoms, *n*[%]	16 [21.05; 16]	11 [45.83; 11]	0.017∗
Composition ratio of death, *n* [%]	14 [18.42; 14]	7 [29.17; 7]	0.190
DLBCL	13 [17.11; 13]	—	—
ENKTCL	—	6 [25; 6]	—
Others	1 [1.32; 1]	1 [4.17; 1]	—

Features of explanation: ^∗^*p* < 0.05 represents statistical differences. GCB subtype: germinal center B-cell subtype; ABC subtype: activated B-cell-like subtype; LDH: lactate dehydrogenase; DLBCL: diffuse large B-cell lymphoma; ENKTCL: extranodal NK/T cell lymphoma, nasal type; PTCL: peripheral T-cell lymphoma; B-LBL: B-lymphoblastic lymphoma; MCL: mantle cell lymphoma; MALT: mucosa-associated lymphoid tissue extranodal marginal zone lymphoma; FL: follicular lymphoma; BL: Burkitt lymphoma.

## Data Availability

All data generated or analyzed during this study are included in this published article.
